# Burnout, Goal Orientation and Academic Performance in Adolescent Students

**DOI:** 10.3390/ijerph17186507

**Published:** 2020-09-07

**Authors:** Pablo Usán Supervía, Carlos Salavera Bordás

**Affiliations:** 1 Departament of Psychology, Faculty of Education, University of Zaragoza, 50009 Zaragoza, Spain; pusan@unizar.es; 2 OPIICS Research Group, University of Zaragoza, 50009 Zaragoza, Spain

**Keywords:** burnout, goal orientation, academic performance, adolescent, students

## Abstract

During their school years, students can have different experiences and go through various emotional and motivational states that can affect their learning experience and play a key role in their personal and academic development. The goal of this paper is to analyse the relationship between goal orientation, burnout and academic performance. **Material and methods:** The study comprised a sample of 2652 students aged between 12 and 19 years (m = 14.55; DT = 1.70), both male (n = 1.368; 51.58%) and female (n = 1.284; 48.41%), from 14 secondary schools. The instruments used were the Perception of Success Questionnaire (POSQ), the Maslach Burnout Inventory—Student Survey (MBI-SS) and academic performance, which was measured using the students’ average school marks. **Results:** Results indicate a significant relationship between task orientation (and, to a lesser extent, ego orientation), efficacy and academic performance in line with adaptive behaviours. In addition, it was demonstrated that task orientation, efficacy and cynicism (burnout) can be used to predict academic performance in adolescents. **Conclusion:** It is argued that goal orientation plays a key role in promoting adaptive behaviours in an academic context and in the personal and academic development of adolescent students.

## 1. Introduction

In recent years, interest in educational research has grown exponentially. The study of psychological variables in students has grown exponentially not only to determine the proper functioning and personal development of students but also to promote their adherence and permanence in the educational system [1].

During adolescence, the use of personal strategies and skills to cope with the challenges and demands of academic life is especially important because this is a vital period for the development of adult personality [2].

In this setting, emotional and motivational experiences play a key role: students face various challenges and may experience sensations and perceptions that can undermine their motivation and commitment, as well as their academic performance, leading them to drop out of school [3].

One of the most relevant cognitive-social theories in the study of school motivation is the achievement goal theory [4,5]. This refers to the goals or reasons that guide students in their academic behaviour in achievement environments, such as school. The students’ main aim is to demonstrate personal skill or ability, and their actions may be guided by two underlying motivational drives: first, a more adaptive behaviour, linked to intrinsic motivation, goal orientation and learning goals, whereby the tasks are undertaken efficaciously and for their own sake. Second, a less adaptive behaviour, linked to extrinsic motivations and performance goals, whereby the tasks are undertaken as a source of gratification and external recognition and validation, and as a way to avoid failure [6].

In this way, the achievement goal theory deals with the motivational orientation of students in achievement environments [5]. Task-oriented students presume that academic success and meeting school demands stem from values such as effort, sacrifice and commitment, and this is related to assuming intrinsic motivations in performing tasks [7], developing coping strategies to face adverse situations [8], effort and academic enjoyment [9] and greater physical and psychological well-being [10]. Conversely, ego-oriented students focus on possessing skills superior to those of their peers, which is closely linked to predominantly extrinsic and non-adaptive motivations [11], less efficacy in performing tasks [12], anxiety, stress, a lack of commitment and possible dropout [13].

Burnout is an individual response to prolonged conditions of stress in a given organisational setting [14]. During their compulsory schooling, some students undergo temporary conditions of stress (of varying levels), which prompt them to lose interest and commitment and to doubt their own ability to meet academic demands. The convergence of all these features is known as school burnout syndrome [15]. This syndrome comprises three dimensions: emotional exhaustion, cynicism and efficacy. Emotional exhaustion is related to various degrees of persistent physical exhaustion and emotional attrition during school years; cynicism is reflected as indifference and lack of interest in school tasks; efficacy reflects the student’s perception of his/her own ability to undertake school tasks.

Academic burnout has been related to low levels of academic engagement and academic self-efficacy [16], poor academic performance [17] and low levels of personal well-being and academic happiness, factors that may lead to school failure [18].

Academic performance can be defined as the quantitative and qualitative assessment of the skills acquired during the learning process [19]. Academic performance is a broad multidimensional concept, which may be measured in relation to academic targets and expectations [20]. Academic performance has been analysed from two main perspectives: a result-focused approach, chiefly based on academic school results; and a more personal approach, which takes into consideration the characteristics of students and their social environment [21]. Quantitative marks are one of the most stable indicators of academic performance [22] but alternative systems have been tried, such as standardised tests, the number of failed subjects and/or repeated courses and even the time invested in studying [23,24,25].

The study of such a broad construct as academic performance has been addressed from a number of perspectives. Portolés and Gónzalez [26] focused on the impact of personality-forming factors on performance; Fierro, Almagro and Sáenz-López [27] examined the impact of different types of motivation and emotional intelligence on academic performance; Guerra and Guevara [28] established several academic variables that affect performance; and Pulido and Herrera [29] focused on the predicting value of socio-demographic factors on performance.

According to Méndez [30], more research is needed to increase our understanding of the different variables that affect performance, to promote the students’ personal development and academic satisfaction and to ensure that they go through their school years successfully, avoiding dropout.

Thus, owing to the lack of specific studies that establish a direct link between the variables at hand, the aim of this study is to analyse the relationship between goal orientation, burnout and academic performance in a sample of adolescent school students.

Three hypotheses are put forth:

**Hypothesis** **1** **(H1).** *Goal orientation is related to efficacy and academic performance, in line with more adaptive behaviours;*

**Hypothesis** **2** **(H2).** *Ego orientation is related to greater levels of physical/emotional exhaustion and cynicism, in line with less adaptive behaviours;*

**Hypothesis** **3** **(H3).** *Goal orientation and personal academic performance can be used to predict academic performance in adolescents.*

## 2. Material and Methods

### 2.1. Sample

The sample comprises 2652 adolescent students aged 12 and 19 years (*m* = 14.55; DT = 1.70), both male (*n* = 1.368; 51.58%) and female (*n* = 1.284; 48.41%), from 14 secondary schools. Inclusion criteria were the ability to read and communicate in perfect Spanish to make sure that they could understand and answer the questionnaire. Incomplete questionnaires were not counted (34) and students with cognitive disorders were excluded owing to their lack of ability to fully understand the questionnaires. The schools were chosen by random sampling and all the students enrolled in the selected schools took part in the study. The percentage of valid questionnaires was 98.72%. The study was carried out in January and February 2020.

### 2.2. Instruments

Three questionnaires were used to measure the variables under consideration.

Achievement goals were measured through the use of the Perception of Success Questionnaire (POSQ) [31] which was previously translated into Spanish and validated by Martínez, Alonso and Moreno [32]. This questionnaire includes 12 items to measure achievement goals with two dimensions: task-oriented (6) (e.g., “When I’m in class, I perform to the best of my ability”); and ego-oriented (6) (e.g., “When I’m in class, I feel successful when I show the teacher and my classmates that I am the best”). Answers express the student’s degree of agreement or disagreement on a Likert-type scale that ranges from “Strongly disagree” (1) to “Strongly agree” (5). The reliability of this questionnaire has been confirmed by several studies conducted in school contexts. It yields Cronbach’s alpha values of 0.85 for the task subscale and 0.82 for the ego subscale and values of 0.85 and 0.84 respectively, in our study.

Academic burnout was measured with the Spanish version of the Maslach Burnout Inventory—Student Survey (MBI-SS) [33]. This questionnaire comprises 15 items distributed in three dimensions: physical/emotional exhaustion (5) (Cronbach’s alpha = 0.82) (e.g., “Studying or going to class all day is exhausting”); cynicism (4) (Cronbach’s alpha = 0.80) (e.g., “I have become less enthusiastic about my studies”); and self-efficacy (6) (Cronbach’s alpha = 0.79) (e.g., “I feel stimulated when I achieve my study goals”). Responses are structured on a Likert-type scale and range from “Completely disagree” (1) to “Completely agree” (5). The original questionnaire yields a Cronbach’s alpha value of 0.80, while in this study it yielded a value of 0.81.

Finally, academic performance was measured using the students’ average marks as expressed in their school reports after the first trimester. It is expressed on a scale from 0 (minimum) to 10 (maximum). The system is widely used and is one of the most stable indicators of academic performance [22,34]. This marking system yields a Cronbach’s alpha value of 0.77 in our study.

### 2.3. Protocol

The students filled out all questionnaires in their own classrooms and during a single day, which was previously agreed with their school’s head of studies. Prior to the study, the schools agreed to participate and the students’ parents/guardians were informed that participation was voluntary, and they signed an informed consent form, in accordance with all the ethical guidelines set out in the Declaration of Helsinki [35]. The study protocol was approved by the Ethics Review Committee of the Psychology and Sociology Department—University of Zaragoza (S46_17R). All questionnaires were anonymous and participation was voluntary. All participants were allowed to drop out of the study at any point, if they so wished.

### 2.4. Data Analysis

Descriptive statistics were used to assess the sample’s socio-demographic background as well as the data and the other variables under consideration. Subsequently, correlations between goal orientation, burnout and academic performance were examined with the aid of the IBM SPSS v26.0 (IBM, Armonk, NY, USA) Stepwise multiple regression was carried out to estimate the predictive value of goal orientation and burnout over academic performance. This procedure allows the generation of a linear model in which the value of the dependent variable (academic performance) is determined from a set of independent variables called predictors (goal orientation and burnout). Finally, a structural equations model was applied using the maximum likelihood method to validate and quantify the causal relations between the three variables (burnout, goal orientation and academic performance) with AMOS v24 software (IBM, Armonk, NY, USA) in order to approach the objective and hypothesis of our study. All the calculations adopted a *p* ≤ 0.05 level of significance and a 95% confidence level.

## 3. Results

### 3.1. Demographics

This study comprised 2652 students, both male (*n* = 1.368; 51.58%) and female (*n* = 1.284; 48.41%), with ages ranging from 12 to 19 years (*m* = 14.55; DT = 1.70) (Table 1).

### 3.2. Descriptive Variables

As illustrated in Table 2, results differed significantly between male and female respondents. Based on Cohen’s *d*, small differences are attested in terms of goal orientation, in which female respondents yield slightly higher results (−0.237). The differences in terms of cynicism, in which males yield higher average values (0.276), are somewhat more pronounced. Concerning the other variables, females yield slightly higher scores than males in physical/emotional exhaustion, efficacy and academic performance, while males yield higher results in ego orientation.

### 3.3. Correlation Analysis between Goal Orientation, Burnout and Academic Performance

The analysis of the variables revealed significant correlations between goal orientation, burnout and academic performance (see Table 3). First, the results indicate strong positive correlations between task orientation and academic efficacy (*r* = 0.512) and academic performance (*r* = 0.282), and strong negative correlations between task orientation and physical/emotional exhaustion (*r* = −0.207) and cynicism (*r* = −0.433). Ego orientation is also strongly correlated with academic efficacy (*r* = 0.248) and, to a lesser extent, with academic performance (*r* = 0.158).

Concerning academic burnout variables, physical/emotional exhaustion (*r* = −0.179) and cynicism (*r* = −0.395) are negatively correlated with academic performance.

Finally, academic performance is positively correlated with academic efficacy (*r* = 0.434).

### 3.4. Regression Analysis of Goal Orientation and Academic Burnout as Predictors of Academic Performance

Multiple regression analysis was conducted, using goal orientation and burnout as predictor variables, and academic performance as a criterion variable. Table 4 shows the steps in the models, with the introduction of the explicative variables which have a significant effect on the likelihood of predicting goal orientation.

As shown, the academic efficacy variable has a direct and significant effect (Step 1). With the incorporation of cynicism (Step 2), the explained variance increased from 42.1% to 47.2%. Finally, the incorporation of task orientation (Step 3) triggered a small increase in the explained variance to 49.2%, leading to a better fit of the model, using academic efficacy, cynicism (negative) and goal orientation. Nagelkerke’s *R^2^* yielded a value of 0.492 for these variables.

### 3.5. Structural Equations Model for Goal Orientation, Burnout and Academic Performance

**Figure 1:**
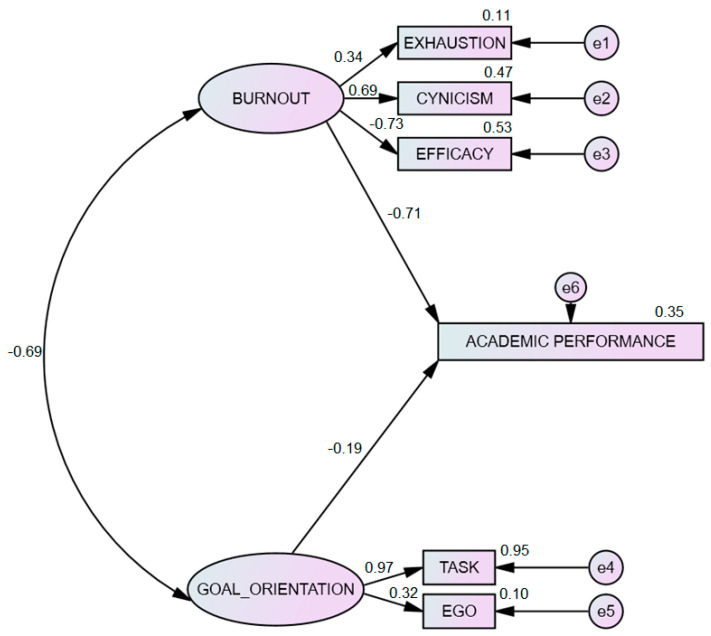
Structural equations model for goal orientation, burnout and academic performance.

Finally, Figure 1 shows the result of the analysis undertaken with structural equations and the maximum likelihood method, which confirms the suitability of the model and the constructs considered herein. The model indicates a negative correlation between academic burnout and academic performance (*r* = −0.71) which suggests that high levels of burnout lead to low academic performance. Goal orientation and academic performance were also found to be correlated (*r* = −0.19). Concerning the fit of the model, the different indices yielded adequate results, confirming the suitability of the factorial structure model proposed to analyse the relationship between goal orientation, burnout and academic performance: χ^2^ (12) = 29.239, *p* < 0.001; χ^2^/gl = 2.436; CFI = 0.96; NFI = 0.96; TLI = 0.92; RMSEA = 0.076, IC 95% (0.048–0.096).

Nested model comparisons were made (Table 5), and assuming the unconstrained model to be correct, we obtained a comparison made with the measurement weights, which indicated that the equal measurement weights model displayed the same fit as the model with no restrictions. Thus, the regression weights in the indicated model were the same. Likewise, the models of the measurement intercepts, structural covariances and measurement residuals did not fit the data, and the comparison indicated that the model statistically differed and gave a worse fit. These results indicated that it was not necessary to include gender differences in the model established among the three variables (goal orientation, burnout and academic performance). 

## 4. Discussion

The aim of this paper is to analyse the relationship between goal orientation, burnout and academic performance in adolescent secondary school students.

H1 linked task orientation and efficacy to academic performance, in line with more adaptive behaviours.

This hypothesis was fully confirmed: the results indicate that a close relationship between these variables exists. As such, task-oriented students work on the basis that academic success comes from effort, interest and motivation, and this is positively correlated with self-efficacy and better school results, in a clear pattern of adaptive behaviour. In addition, task orientation was found to be negatively correlated with physical/emotional exhaustion, cynicism and burnout.

Various studies have pointed out, partially or fully, the relationship between task orientation, burnout and performance. Baños, Ortiz, Baena and Tristán [36], analysing a sample of secondary school students, found that intrinsically task-oriented students also presented higher levels of self-efficacy, leading to better academic results. Cuevas, García and Contreras [37] related task orientation to higher levels of satisfaction and enjoyment, as well as greater effort and interest in performing tasks. Usán, Salavera and Teruel [38] linked task orientation to intrinsic motivations, as well as better academic results. Suárez and Suárez [39] indicated the influence of goal orientations on the adoption of learning strategies that lead to higher levels of self-efficacy. Finally, Correa, Cuevas and Villaseñor [40] referred to a profile of adaptive behaviour, characterised by task orientation and better academic results, as well as a higher degree of psychological well-being.

H2 linked ego orientations to physical/emotional exhaustion and cynicism, in line with less adaptive behaviours.

This hypothesis was not confirmed: not only did the results of the study not reveal a significant correlation between these three variables, they revealed a correlation between them and efficacy and academic performance, although this correlation was less pronounced than that attested with task orientation.

These results imply that ego orientation is not necessarily related to exhaustion and cynicism. According to Saies, Arribas, Cecchini, De Cos and Otaegi [41], ego orientation is not always related to non-adaptive behaviours. High cognitive skills can help subjects to choose different elements from both types of goal orientation in order to undertake their academic duties.

On the other hand, as pointed out by Jaramillo [42], students can combine both orientations without contradiction and obtain good academic results, regardless of which orientation is predominant, suggesting a natural selection between two orthogonal choices [5].

Finally, in line with our results, Barbosa, Tristán, Tomás, González and López [43] found no relationship between extrinsic goal orientation and the different dimensions of academic burnout. Other studies argued that task orientation is more strongly correlated with academic performance than ego orientation [44,45].

Therefore, students can strike a balance between both types of goal orientation, depending on their academic demands and interests and their individual perspective [46].

Our H3 alluded to the predictive value of task orientation and academic efficacy over the academic performance of adolescent students.

This hypothesis was partially confirmed by our results; although it was demonstrated that efficacy and task orientation can predict academic performance in the third step of our regression model, the second step yielded a negative value for cynicism, along with a high percentage of the explained variable.

The results suggest that these variables can be used to predict academic performance. There is consensus in the existing literature concerning the fact that high-performing students yield higher values in both types of goal orientation than low-performing students [39,47], although the studies that have investigated the predictive value of goal orientation over academic performance have not yielded clear results [48].

In a similar vein, various studies have pointed out the predictive value of efficacy over academic performance. Coschiza, Martín, Gapel, Nievas and Ruiz [49] analysed the socio-demographic variables that affect school performance, including self-efficacy; Gamazo, Martínez, Olmos and Rodríguez [50] assessed a series of factors related to efficacy, including academic performance; Luzarraga, Nuñez and Etxeberria [51] linked high-efficacy students to good academic results; and, finally, Vizoso and Arias [52] emphasised the relationship between these variables in a study about academic stress factors and the well-being of students.

At any rate, the impact of the variables being studied on the personal and academic development of the students is clear. Along with other personal and contextual circumstances, they confirm the psychological and academic setting within which the students’ school experience is framed. This has a direct effect on the students’ commitment and, with it, on the likelihood of dropout and the students’ personal and academic development [53].

## 5. Conclusions

Our results have direct implications for educational strategies; teachers and other members of the school community ought to promote self-determined attitudes such as academic commitment, interest and motivation from an early age, in order to help students feel efficacious in performing their tasks, leading to greater persistence and dedication. This may also contribute to prevent poor academic performance and improve their personal and academic development.

Programmes directed by educational professionals and schools can help the students to improve their school experience, contributing to the comprehensive development of students, preventing school dropout and improving their academic performance.

Our results encourage us to continue investigating, incorporating new psychological and academic variables and seeking new questions with which to define methodologies to progress the personal and academic development of adolescents.

### 5.1. Limitations of the Study

The limitations of this study arguably lie in its cross-sectional design since data were collected at a given moment in time. The scores can change from one year to the next and even within the same school year, depending on the students’ personal and contextual circumstances, despite the large size of the sample. In addition, although measuring academic performance quantitatively is the most uniform and widespread method, students’ marks are likely to oscillate from one year to the next, and even from one trimester to the next. Similarly, the secondary schools approached for the survey were randomly selected and are not representative of the city’s social structure. Therefore, results may be affected by socioeconomic bias or other factors, such as different levels of education.

### 5.2. Future Prospects

In the future, it would be fruitful to implement longitudinal models to evaluate the evolution of the constructs under analysis over time. This, however, poses the additional logistic challenge of tracing each subject over several periods. It would also be interesting to take into consideration other educational tiers, such as primary schools and universities. In addition, it would be interesting to attend to the mediating role of academic burnout between goal orientations and academic performance or even other psychological variables in students. Similarly, it would be interesting to consider other related variables, such as socio-demographic features, such as gender, school year, the type of schools and other social and personal variables such as socio-economic status.

## Figures and Tables

**Table 1:** Socio-demographic data of the sample.

		N	%
**Gender**	Male	1368	51.58
	Female	1284	48.41
**Age**	12 years	342	12.89
	13 years	368	13.87
	14 years	427	16.10
	15 years	475	17.91
	16 years	502	18.92
	17 years	456	17.19
	18 years	68	2.56
	19 years	14	0.52
**School year**	1º ESO	416	15.68
	2º ESO	510	19.23
	3º ESO	638	24.05
	4º ESO	652	24.58
	1º BACH	311	11.72
	2º BACH	125	4.71
**Repeating course**	Yes	596	22.47
	No	2056	77.52
**Type of school**	Public	1968	74.20
	Private	644	25.79

**Table 2:** Results of goal orientation, burnout and academic performance.

	Total		Males		Females		
	*x*	*sd*	*x*	*sd*	*x*	*sd*	*Cohen’s d*
**Goal orientation (task)**	3.79	0.80	3.70	0.83	3.89	0.77	−0.237
**Goal orientation (ego)**	2.93	1.02	2.98	0.96	2.88	1.08	0.097
**Physical/emotional exhaustion**	3.23	0.97	3.20	1.03	3.26	0.89	−0.062
**Cynicism**	2.16	1.06	2.30	1.08	2.01	1.02	0.276
**Academic efficacy**	3.54	0.75	3.52	0.79	3.56	0.71	−0.053
**Academic performance**	6.24	1.14	6.22	1.13	6.26	1.15	−0.035

**Table 3:** Correlation analysis between goal orientation, burnout and academic performance.

	1	2	3	4	5	6
**1. Goal orientation (Task)**	1					
**2. Goal orientation (Ego)**	0.308 **	1				
**3. Physical/emotional exhaustion**	−0.207 **	0.011	1			
**4. Cynicism**	−0.433 **	−0.059	0.357 **	1		
**5. Academic efficacy**	0.512 **	0.248 **	−0.172 **	−0.491 **	1	
**6. Academic performance**	0.282 **	0.158 **	−0.179 **	−0.395 **	0.422 **	1
*Average*	3.79	2.93	3.23	2.16	3.54	6.24
*DT*	0.80	1.02	0.97	1.06	0.75	1.14
*Alfa de Cronbach*	0.85	0.84	0.80	0.79	0.81	0.77

** The correlation is significant at 0.01 (bilateral).

**Table 4:** Regression analysis of goal orientation and burnout as predictor variables of academic performance.

		*B*	s.e.	*R^2^*	*t*	Sig.
**Step 1**	**(Constant)**	0.860	0.195	421	4.416	0.000
	**Academic efficacy**	0.640	0.054		11.907	0.000
**Step 2**	**(Constant)**	2.075	0.272	472	7.628	0.000
	**Academic efficacy**	0.458	0.060		7.627	0.000
	**Cynicism**	−0.263	0.042		−6.221	0.000
**Step 3**	**(Constant)**	1.965	0.277	492	7.105	0.000
	**Academic efficacy**	0.425	0.062		6.867	0.000
	**Cynicism**	−0.269	0.042		−6.376	0.000
	**Goal orientation (Task)**	0.082	0.039		2.073	0.039

Variables excluded: physical/emotional exhaustion (burnout)/goal orientation (ego).

**Table 5:** Multiple-group analysis of goal orientation, burnout and academic performance by gender.

	DF	CMIN	*p*	NFI Delta-1	IFI Delta-2	RFI rho-1	TLI rho-2
**Measurement weights**	5	11.182	0.048	0.014	0.014	−0.033	−0.034
**Measurement intercepts**	11	37.970	0.000	0.047	0.048	−0.035	−0.036
**Structural covariances**	14	39.485	0.000	0.049	0.050	−0.051	−0.053
**Measurement residuals**	20	55.563	0.000	0.069	0.070	−0.061	−0.064
